# Developing capacity for learning community systems: Experiences from the 100 Million Healthier Lives SCALE Initiative

**DOI:** 10.1002/lrh2.10296

**Published:** 2021-11-17

**Authors:** Tara Carr, Margaret Holly, Kristin Reed, Rumana Rabbani, Caroline Chandler, Brittany Cook, Paul Howard, Marianne McPherson, Becky Henry, Rohit Ramaswamy

**Affiliations:** ^1^ University of North Carolina at Chapel Hill Chapel Hill North Carolina USA; ^2^ Wandersman Center Columbia South Carolina USA; ^3^ Institute for Healthcare Improvement Boston Massachusetts USA; ^4^ Pueblo Department of Public Health and Environment Pueblo Colorado USA

**Keywords:** causal loop diagrams, community transformation, experience, meta‐ethnography, participatory action synthesis

## Abstract

**Introduction:**

This paper explores the capabilities that contribute to community transformation and the common pathways followed by communities in the 100 Million Healthier Lives SCALE (Spreading Community Accelerators through Learning and Evaluation) initiative in their transformation journeys towards a “Culture of Health”.

**Methods:**

Funded by the Robert Wood Johnson Foundation (RWJF), from 2016 to 2020, between 18 to 24 community coalitions nationwide participated in SCALE, the goal of which was to co‐design, implement, test, and scale up a model called the Community of Solutions (COS) Framework, that built community capacity around a set of skills and behaviors to advance culture change and create sustainable improvement in health, well‐being, and equity. We adapted and applied two qualitative research techniques, meta‐ethnography and participatory action synthesis, to evaluate SCALE initiative data.

**Results:**

Eight concepts emerged that represent the knowledge, capabilities and practices commonly acquired and utilized across the communities. Overall, these concepts emphasize individual and team leadership, quality improvement skills, an intentional focus on equity, and partnerships for spread and sustainment. Concepts were linked into lines of arguments which were unique storylines explaining the transformation pathways. Three stories of the transformation process emerged from the data. Causal Loop Diagrams (CLDs) were created to represent non‐linear system relationships and visually capture some of the most important dynamics of the process of transformation. Even with vast heterogeneity among the SCALE communities and the diversity of activities that the communities undertook, our analysis showed there were a few basic principles that undergirded the process of building capability for transformation.

**Conclusions:**

The knowledge from our findings should be useful to expand further research and practice in community learning systems.

## 1 INTRODUCTION

### 1.1 Background

In 2014, Dr Risa Lavizzo‐Mourey, then CEO of the Robert Wood Johnson Foundation, laid out a bold new strategy for the foundation, based on the paradigm of a “Culture of Health.” This paradigm envisions the attainment of population health, well‐being, and equity through cross‐sectoral collaborations between organizations responsible for healthcare, social services, education or economic development, and community coalitions. The need for systems changes at the community level, and the urgent imperative to focus on equity has never been more apparent than during the devastation from COVID‐19, where a thousand different versions of the pandemic have been manifested in a thousand different communities. Public health analysis and responses to COVID‐19 have not adequately considered the effect of inequities arising from the intersection of race, gender, and class on an individual or community's experience of the pandemic.^1^, ^2^

The COVID‐19 pandemic has merely reinforced what has been known for a long time. Health and well‐being are intimately connected to community level contexts and require collaborative, transformational solutions that are built on communities' capacity to learn, in real‐time, how to improve what matters most to them.^3^ In this paper, we present the implementation, evaluation, results, and learning from a multiyear, multisite project called SCALE (Spreading Community Accelerators through Learning and Evaluation), part of the 100 Million Healthier Lives Initiative, that focused on building the capacity of community coalitions to develop relationships and partnerships and learn methods to transform health, well‐being, and equity. Using a detailed description of the implementation process, we describe the most common pathways that communities followed in their transformation journey, the knowledge, capabilities, practices and relationships that were used, and the constraints and barriers that communities faced. The knowledge from our findings should be useful to expand further research and practice in community learning systems.

### 1.2 Context: The 100 Million Healthier Lives (100MLives) SCALE Initiative

Convened by the Institute for Healthcare Improvement (IHI), 100MLives was a global movement and collaboration from 2014 to 2020 in support of Robert Wood Johnson Foundation's Culture of Health (COH) Strategy.^4^ SCALE, the flagship initiative under 100MLives, was a learning collaboration that initially was cofacilitated by four organizations working on health and healthcare improvement—IHI, Communities Joined in Action, Community Solutions, and the Network for Regional Healthcare Improvement—together with community coalitions selected through a competitive application process. The goal of SCALE was to co‐design, implement, test, and scale up a model called the *Community of Solutions (COS) Framework* that built community capacity around a set of skills and behaviors to advance culture change and create sustainable improvement in health, well‐being, and equity.^5^, ^6^ The four organizations trained and supported the communities in their improvement efforts. SCALE communities applied the COS model to a variety of topics including healthy food access, infant mortality, refugee education and health, chronic disease, self‐management, youth nutrition and physical activity, homelessness, access to public parks and green space, and youth development and education. Regardless of topic areas of focus, all SCALE communities agreed to apply an equity lens to the work. During the first phase of the initiative from 2016 to 2018, 24 community coalitions were part of SCALE. Eighteen stayed on in the second phase from 2018 to 2020.

### 1.3 Questions of interest

Our description of SCALE in this paper focuses on the following four questions:

1. What are the key contributors to achieving capability for community transformation?
2. What knowledge, capabilities, practices, and relationships did communities acquire and utilize?
3. What common pathways did communities follow in their transformation journey?
4. What are the mechanisms through which communities brought about change?

## 2 METHODS

### 2.1 SCALE theory

**FIGURE 1:**
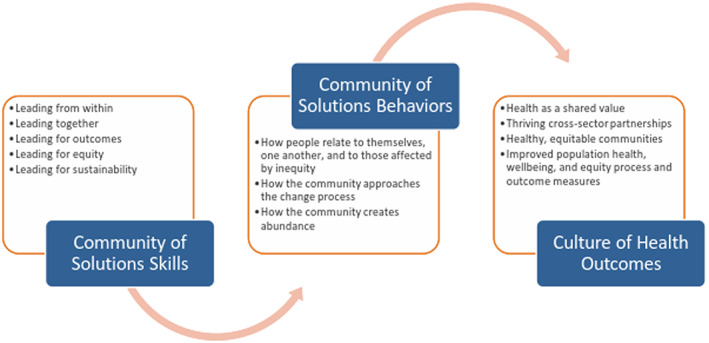
The SCALE project's COS model

The theory of change for SCALE is the COS model shown in Figure 1. These elements of the model were influenced by the following literature on community capacity building and adapted for SCALE based on practical experience of the implementing partners. The community problem solving and change framework^7^, ^8^ specifies the need to build operational and problem solving capacities of communities to enable them to engage in comprehensive change strategies. Zakocs and Edwards^9^ and Zakocs and Greenberg^10^ describe leadership style, member participation, group cohesion, participatory decision‐making, and involvement of local government as key characteristics for successful coalition effectiveness and capacity. Flasphohler et al^11^ emphasize the need to build different types of capacity (general vs innovation specific) at multiple levels (individual and community). Research from the field of implementation science posits that while training is necessary to develop skills, training alone is not enough to achieve outcomes and that training needs to be reinforced through technical assistance (eg, coaching and problem solving), tools, and iterative quality improvement processes.^12^

### 2.2 SCALE intervention components

The primary activities based on the COS model is the development—co‐designed with communities—of skills of community coalition members in five areas (personal growth and leadership; working with others; design, improvement and implementation science; centering equity; and long term planning) through intense training, coaching and support activities. The key activities are shown in Table 1. The types of coaching calls are outlined in Table 2.

**TABLE 1:** Key activities

1	3‐d Community Health Improvement Leadership Academies (CHILA) (learning, action and community‐building sessions)
2	Monthly network calls—to provide additional training and support, share progress and challenges, highlight the work of one SCALE community and co‐design next steps for the SCALE initiative
3	Monthly coaching and peer support in regional networks (3‐4 communities per network) in which coaches and other SCALE communities provided coaching and support to each other to address challenges and share best/promising practices
4	Monthly individual coaching and support—customized to meet the needs of each SCALE community
	Specialty coaching and support as needed/requested
6	Quarterly milestones with each SCALE communities to review progress, address challenges and develop plans for the next 90 days
7	1‐d on‐site meeting with the implementation team for each SCALE community to refine their measurement plans, theory of change and/or other elements to accelerate their work
8	Training on planning and running an equity action lab‐equity action labs provide a highly adaptable structure and strategy to bring together a diverse team to make meaningful progress on a complex goal in a short amount of time (generally 100 d).^13^
9	A continually refined and streamlined system to continually track progress, assess general capability and use this data for improvement.

**TABLE 2:** Coaching call summary

Coaching call summary (explained further below)				
Call name	For	Facilitated by	Length	Number and frequency
Multi‐Regional Network Calls	SCALE 1.0 Local Improvement Advisors for each Multi‐Regional Network	SCALE Coaches	1 h	5 per month (one per Multi‐Regional Network) 1 call/month for each coach
SCALE 1.0 Community Calls	SCALE 1.0 Local Improvement Providers from each SCALE 1.0 Community	SCALE Coach	1 h	18 per month (one per SCALE 1.0 Community) 3‐4 calls/month for each coach
Regional Network Calls	SCALE Spread Communities within a Regional Network	SCALE 1.0 Community	1 h	18 per month (one per Regional Network) starting in Phase 2 3‐4 calls/month for each coach
SCALE Spread Individual Community Calls	Each SCALE Spread Community	SCALE 1.0 Community	1 h	72‐108 calls per month (one per SCALE Spread Community participating in a Regional Network or 4‐6 calls per Regional Network)
Milestone Calls	SCALE 1.0 Communities full team	SCALE Implementation Team	1 h	18 calls per quarter 3‐4 for each coach
Improvement Coach Professional Development Program Calls	SCALE 1.0 Team members participating in this program	IHI Improvement Advisors	1.5‐3 h	3‐6 Virtual sessions between September and December 3‐6 for each coach; coaches will participate in the program
Community Champions Calls	SCALE 1.0 Community Champions and Spread Community Champions	Ziva Mann and Shemekka Coleman	1 h	1 Per Month (may need to split up by Multi‐Regional Network in Phase 2) Coaches as needed
Specialized Coaching Calls	Depends on the nature of the call	Specialized Coaches	Varies	Varies (specialized coaching is by request) Coaches as needed
Coaches Call	SCALE Coaches	SCALE Implementation Team and representatives of the SCALE Evaluation Team	1.5 h	Monthly

### 2.3 SCALE evaluation

The primary intent of the evaluation was to advance knowledge about the *processes* of change in the complex environments within which the communities operated, rather than attempting to assess the extent to which these processes resulted in the achievement of specific outcomes. The evaluation was intentionally designed as a participatory partnership between the funder, the implementation partners, the communities, and a multidisciplinary evaluation team led by the Universities of North and South Carolina. Recognizing the heterogeneity in coalition structures, community contexts, and areas of improvement emphasis, the evaluation approach was designed to be flexible, adaptive, and process focused. The evaluation also needed to be sensitive to the fact that much of the knowledge about the implementation was generated by and existed within the communities, and that their interpretation of the data was an important part of the knowledge synthesis process. The approach was related to learning or developmental evaluation^14^, ^15^ that are iterative methods using rapid learning cycles to improve implementation processes. The evaluation was segmented into a formative and a summative evaluation component. Both have been described elsewhere and a summary of each is presented here.

### 2.4 Formative evaluation: Learning for improvement

The formative evaluation approach is described in detail in Scott et al.^16^ It was focused on the skills component of the theory of change and was designed as a learning system for ongoing adaptation of the program. Three data collection methods: *inquiry*, *observation*, and *reflection* were used to obtain data about the implementation to capture as much contextual information as possible. Together, these methods provided a multifaceted view of the SCALE implementation process that was synthesized and periodically shared with the implementation team and the communities to stimulate improvements to the program design and its execution.

### 2.5 Summative evaluation: Collaborative meaning making

The summative evaluation was designed in the latter half of the project after the theory of change and the primary components of the program design had stabilized. This evaluation focused on the behaviors component of the theory of change through a longitudinal analysis of each community's traversal through SCALE. Since one of the goals of the summative evaluation was to generate insights from a plurality of voices, a collaborative bottom‐up methodology called ToAST (Telling our Amazing Stories Together) was developed.

Details of the ToAST approach are described in Reed et al.^17^ The approach adapted two qualitative research techniques: meta‐ethnography, a structured seven‐phase method for synthesizing findings from a small number of ethnographic studies to create new interpretations^18^ and participatory action synthesis (PAS),^19^ a communal synthesis process where the researchers bring their own social, political, and cultural experiences to the table. In the version utilized for SCALE, the “ethnographies” were the patchwork of routinely collected project data that each community brought to the synthesis workgroup, and the objective was to weave this patchwork together into a shared story of a transformation journey. The communities involved and their stories can be found at this publicly available site: Community Commons http://www.communitycommons.org/entities/ac745b94-1b33-4143-acc3-6df3e0217b3c

To implement the ToAST approach, a synthesis workgroup consisting of volunteer participants from the SCALE communities and selected implementation and evaluation team members was assembled. The synthesis workgroup systematically worked through the seven phases of meta‐ethnography, working in four topical areas that roughly aligned with the skill areas in the theory of change: *improvement science and measurement, COS skills (personal and team leadership)*, *spread and scale up, race, racism and equity and engaging people with lived experience*. Across communities, the topical sub groups identified similar ideas that were categorized into *themes*. Subsequently, the subgroups contextualized the topic‐specific themes into community‐specific *concepts* representing the workgroup's interpretation of the particular elements of each community's journey through SCALE. For communities that had complete data on all the topical areas, the community‐level concepts were then assembled into *overarching concepts* representing the most common mechanisms employed by all communities to bring about change. Descriptions were developed for each of these concepts and were sent back to the coalition members to validate and revise if needed. Communities were also invited to create *lines of arguments*. In meta‐ethnography, these are new story lines or overarching explanations arising from the synthesis of the date. To create lines of argument, communities linked the overarching concepts visually into transformation pathways. The evaluation team used these to develop causal loop diagrams (CLDs) to highlight the nonlinear nature of the transformation process. Table 3 summarizes the seven phases of meta‐ethnography and how they were applied to the evaluation.

**TABLE 3:** Phases and application of meta‐ethnography

Meta‐ethnography phase	Application to the ToAST process
1. Getting started	Forming the synthesis team
2. Deciding what is relevant to the initial interest	Developing topical area sub‐groups
3. Reading the studies	Each community's interpretation of its data
4. Determining how the studies are related	Developing sub‐group level themes
5. Translating the studies into one another	Contextualizing themes into community specific concepts
6. Synthesizing translations	Assembling community specific concepts into overarching concepts
7. Expressing the synthesis	Developing lines of argument and CLDs

## 3 RESULTS

We describe results from both the formative and summative evaluations, organized by the questions of interest presented earlier in this paper.

### 3.1 Key contributors to achieving capability for community transformation

The formative evaluation revealed four key areas that were critical drivers for achieving the goals of SCALE: (a) a *robust theory of change* that could serve as a common roadmap for all communities despite heterogeneity in contexts and areas of improvement focus; (b) a *structured improvement process* using a well‐known quality improvement method called the Model for Improvement^20^; (c) a planned *implementation support system* for coaching communities through the process of improvement and change; and (d) an *evaluation approach* that provided ongoing data for learning and improvement. The existence of these areas was necessary but not sufficient. There needed to be a process that could facilitate the adaptation of these areas in response to changes in context or to new and emergent priorities. The process established for feedback of the formative evaluation results provided the mechanism to do this. An example of change to each area based on recommendations from the formative evaluation is shown in Table 4. Detailed results from the formative evaluation are presented in Scott et al.^16^

**TABLE 4:** Change based on formative evaluation

Key area	Example of change based on formative evaluation
Theory	The Theory of Change was modified to include equity as a key driver
Improvement process	An intervention called the “Action Lab” that convened community members with the power to enact change was introduced to improve implementation quality and meaningfully advance equity in a defined time period
Support system	Guidance on deadlines and expectations for coaches was strengthened
Evaluation	As SCALE implementation matured, evaluation resources shifted from monitoring the support system to assessing community‐level implementation

### 3.2 Knowledge, capabilities, practices, and relationships acquired and utilized by communities

The overarching concepts from the ToAST process represent the knowledge, capabilities, and practices commonly acquired and utilized across the SCALE communities. These are shown in Table 5 along with the theory of change components to which these are connected, ordered by the progression of skills from the individual to the community, and by behaviors from relationships to capability for change. Overall, these concepts emphasize *individual and team leadership*, *quality improvement skills*, an *intentional focus on equity*, and *partnerships for spread and sustainment*. The first two concepts (nos. 1 and 2) related to individual leadership skills that gave the SCALE coalition members the confidence to approach complex problems and to engage in sensitive and difficult conversations. The next two (nos. 3 and 4) pertained to capabilities related to team leadership and practices that brought together those with local context specific knowledge needed to bring about change. Concepts 5 and 6 involved the acquisition of technical skills that were needed to complement local knowledge. A systematic approach to improvement based on a change theory, and accessing specialists (eg, improvement coaches) were practices utilized by the communities. Finally, as indicated by concepts 7 and 8, each community made equity and racism a central focus of their work and built deep relationships with other SCALE communities to support and sustain their work.

**TABLE 5:** Concept and theory of change

Concept no.	Concept label	Concept definition	Theory of change component	Plain language explanation
1	Applying a theory of change to guide community efforts	The community first develops and then applies an explicit theory of change (TOC), whereby it conceptualizes specific ideas needed for change to direct its efforts toward community health and well‐being improvement, create a transformational plan, and spread effective strategies to other communities	Leading for outcomes skills influencing how the community affects the change process	The theory of change is a description of the intermediate outcomes that need to be achieved along the way to transformation. This helps communities develop a roadmap of how they will approach their transformation efforts
2	Embedding people with lived experience into transformation work	The community engages people with lived experience in a number of roles, including as community champions, project leaders, trainers, organizers, key informants, and participants throughout the course of the change process	Leading together skills influencing how people relate to one another	Each community was required to select a community member to be part of its leadership team for the project
3	Building capabilities for change community by identifying and growing leaders	The community builds capability of community members to address complex community structural issues that are barriers to community well‐being	Leading from within skills influencing how people relate to themselves	Selected individuals from the community were trained to be leaders who had the confidence to ask difficult questions about the barriers to community change
4	Building the capability of the core team engaged in transformation to engage in peer learning	A community works with partners as a coalition to more effectively direct its improvement efforts. Partners include people that have intimate knowledge of and/or experience in the community as residents, advocates, or through community‐based organizational affiliations	Leading together skills influencing how the community approaches the change process	Organizations that typically may not work with each other but whose collaboration is important were brought together to learn from each other
5	Creating the atmosphere for authentic dialog within and between communities	The community leaders develop relationships and engage community members to create space for, and improve ability to have, difficult or sensitive conversations	Leading from within skills influencing how people relate to one another	Members of the community coalitions felt that they trusted each other enough to have honest and open conversations about how they felt in their relationships with one another
6	Explicitly and intentionally addressing racism and inequity within the community	The community makes efforts to identify and address the systems, policies, and practices working within the community that reinforce structural racism and contribute to disparities and inequities	Leading for equity skills influencing how the community relates to those affected by inequity	All communities were required to examine and acknowledge the effects of racism and inequity on well‐being
7	Creating access to those with specialized knowledge (eg, in QI) for coaching and technical assistance	The community is proactive and intentionally uses support from specialists with topic specific and community‐relevant knowledge	Leading for outcomes skills influencing how the community approaches the change process	Recognizing that there are specific technical skills (eg, quality improvement, leadership, data collection, evaluation) that may not be available within the community, the project team ensured access to external people with these skills
8	Facilitating the formation of personal relationships and social connections across coalitions	The community forms personal relationships with peer communities and provide and receive support to one another to discuss and problem‐solve common community challenges	Leading for sustainability skills influencing how the community creates abundance	There was an active effort to create opportunities for coalition members to build deep and lasting relationships across communities

These eight cross community concepts emerged in Phase 6 of the ToAST process as the output of multiple levels of synthesis shown in Table 3. The earlier phases of this process captured data on the specific knowledge, tools, and relationships at the community level.

### 3.3 Common pathways that communities follow in their transformation journey

**FIGURE 2:**
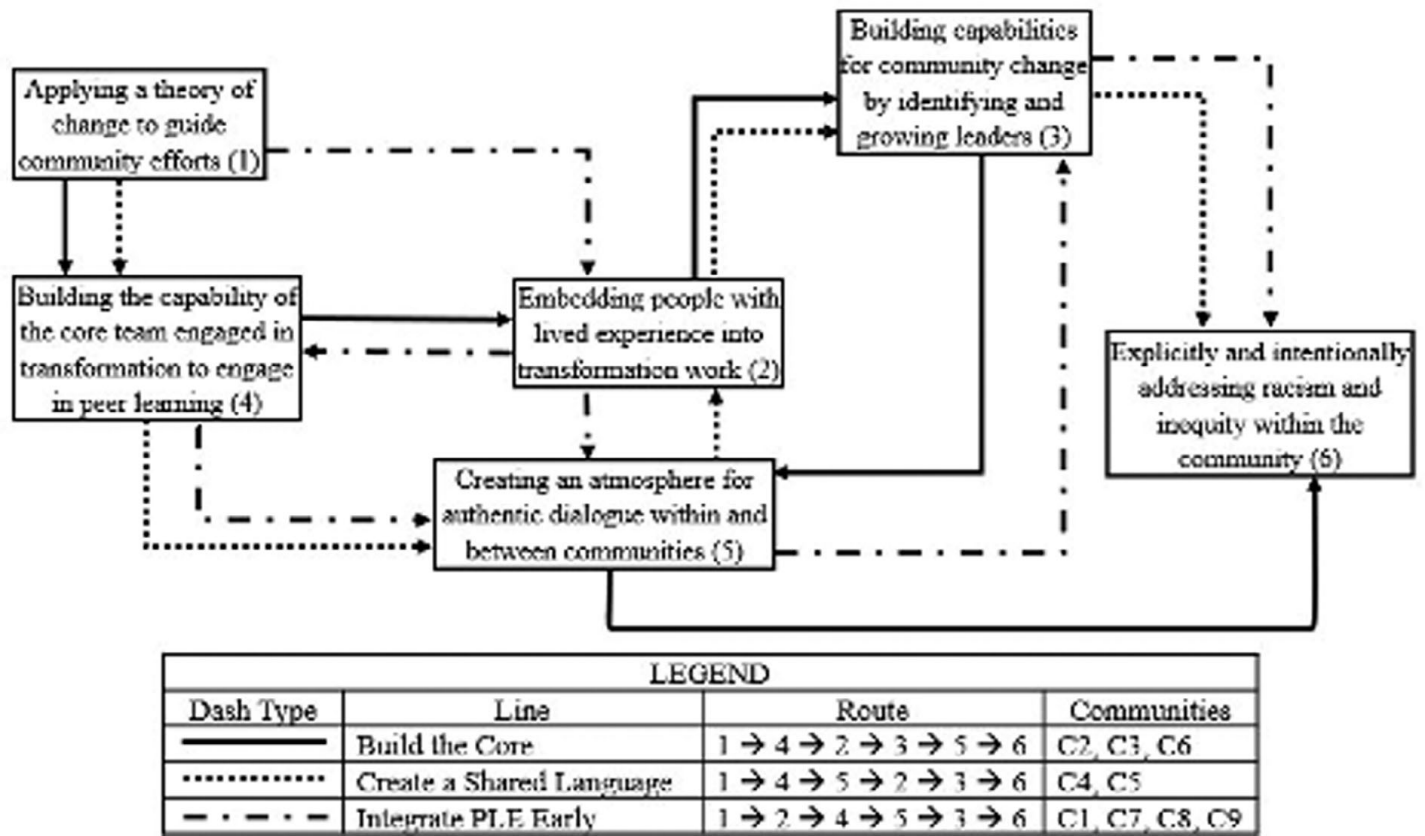
Transformation pathways

The transformation pathways shown in Figure 2 were developed in Phase 7 of the ToAST process by linking six of the eight concepts in Table 5 into lines of argument that are unique storylines explaining how transformation occurs. The other two concepts (forming personal relationships and accessing experts) were clearly necessary capabilities, but were seen by communities as being ongoing, and not on a pathway of change. Each storyline begins and ends at the same concept. All SCALE communities began their transformation process with an explicit theory of change about how they were going to approach building their capacity to address structural racism and inequity that are foundational barriers to community well‐being. Addressing inequity was the “price of admission” into the SCALE initiative, and was the non‐negotiable lens through which transformation efforts were viewed. However, between these two points, different communities emphasized different concepts at different stages of their journey, depending on their particular context and circumstance. Three stories of the transformation process emerged from the data.

The “build the core” story was reported by the coalitions that had strong existing connections with people with lived experience in their communities but first focused on strengthening their internal capacity by learning and internalizing improvement and relationship building tools and then used these to expand and deepen their relationships with the community to spearhead honest and authentic conversations about race and inequity. A core team member of a SCALE community that followed this pathway stated:

*It took longer than expected to develop the infrastructure of the “Quarterback Organization” and to provide engagement opportunities with activities. [Our SCALE community] was focused on fixing the lake and not fixing the fish, which involves more long‐term changes*.

The “create a shared language” story was followed by community organizations that began with using community partners with deep connections within the community to develop a shared language that facilitated mutual communication and dialog. Initial conversations to strengthen the ability to communicate was seen as especially important by these communities as was the need to create safe spaces for open and honest discussions and sharing experiences. These efforts accelerated trust and relationships in these communities. A coalition member stated:

*We had a discussion on racism at every coalition and staff meeting. We created a safe and open space to discuss personal &/or patient experiences with racism or current events dealing with racism*.

While all coalitions had a community champion from the outset, the “integrate people with lived experience (PLE) early” story emphasized developing community leaders as the first component of building the core team's ability to engage with the wider community. They also used the power of the local social networks to build relationships to build relationships quickly to advance equity work.

As one core team member remarked:

*A focus on authentic engagement of partners, building trust and relationships was foundational to the ability to scale up and sustain work. SCALE helped us offer more leadership development opportunities for community champions and staff members. This was valuable for building local capacity to improve authentic engagement and peer mentorship opportunities to lead civic engagement and service leadership*.

### 3.4 Mechanisms through which communities brought about change

**FIGURE 3:**
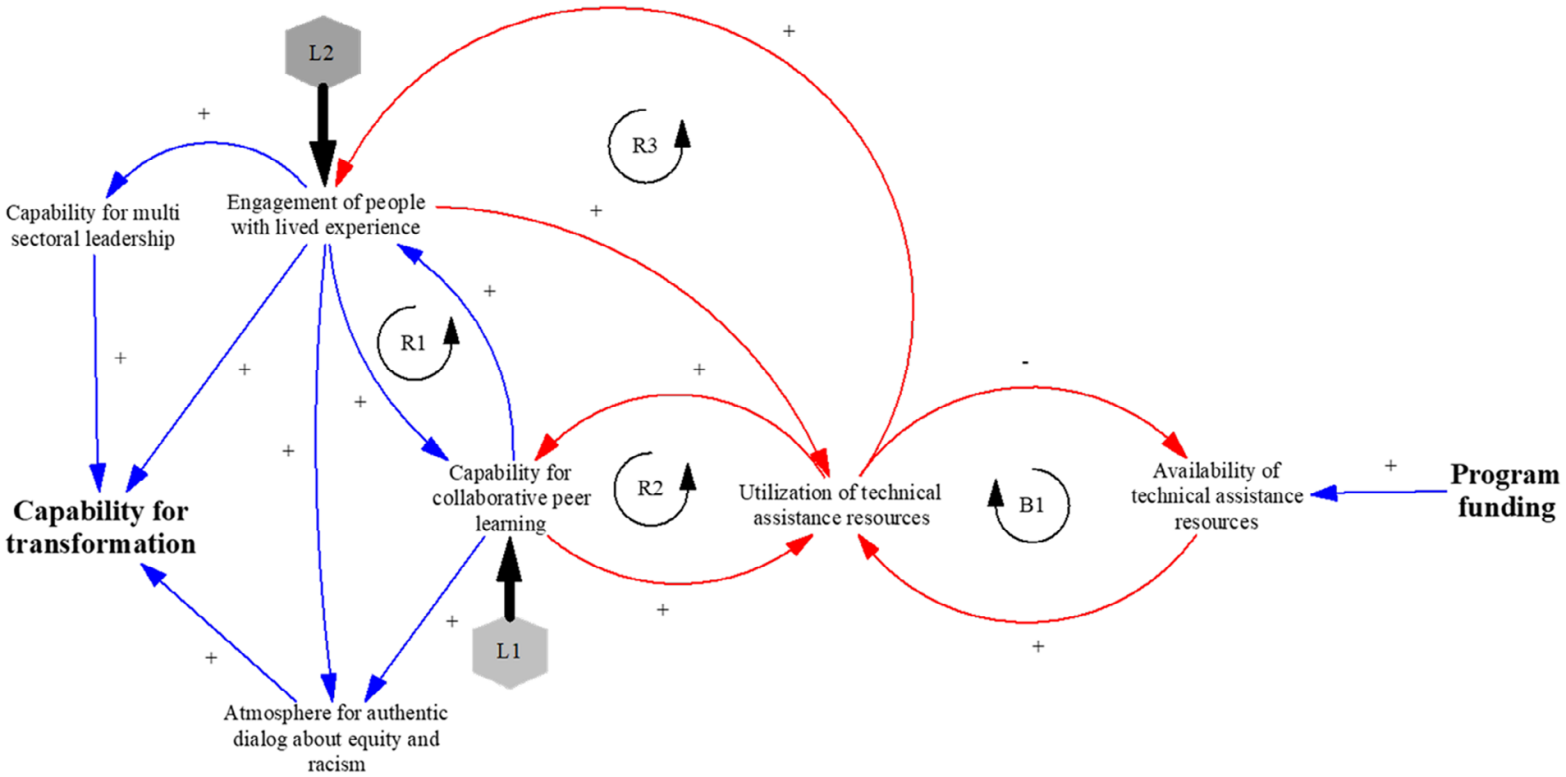
CLD of SCALE transformation pathways

While the lines of argument are a convenient visual representation of how change happens, their sequential structure does not capture the complexity of the change process. CLDs allow us to better represent nonlinear system relationships. Using the data from Figure 2, the evaluation team developed the CLD in Figure 3 to visually capture some of the most important dynamics of the process of transformation.

In Figure 3, the blue arrows represent system characteristics that support transformation, while the red arrows represent constraints to transformation. The positive signs in the loops indicate that the variables move in the same direction: for example, the greater the relationships, the greater the collaborations. The *input* into the system is the available external funding that influences the extent to which SCALE activities can be supported. The *output* from the system is the SCALE goal, that is, the capability for community transformation. As seen from the picture, there are three direct drivers of the output represented by the lines leading to the community transformation box: (a) the ability of communities to create collaborations with broad multisector leadership (including community members); (b) creating opportunities for dialog about addressing racism and equity and (c) engaging people with lived experience.

SCALE program activities were intended to strengthen these drivers. The two places in the system where SCALE interventions were focused (leverage points to change the system) are indicated by the two arrows L1 and L2. L1 indicates that all SCALE training and support activities revolved around strengthening collaboration within the community. This enabled communities both to create leaders from within the community (loop R1) and facilitate dialog around difficult questions about inequity and race, without which true change is not possible. At the same time, to ensure that collaboration did not exclude those who needed to be at the center of transformation activities, SCALE required the intentional engagement of people with lived experience in leadership roles (leverage point L2), which strengthened the capability for transformation in multiple ways.

As SCALE progressed and the community coalitions took on more complex projects, and attempted to spread what they had learned across the region, there was an increasing need for implementation support and technical assistance as indicated by the loops R2 and R3. These required increased resources, which were limited, by the available resources as shown in loop B1 (the more resources get utilized, the less remains available). SCALE communities needed to do their best with fixed resources that were stretched thin, and these served as a limit to what the communities could ultimately achieve.

## 4 DISCUSSION

Even with vast heterogeneity among the SCALE communities and the diversity of activities that the communities undertook, our analysis showed there were a few basic principles that undergirded the process of building capability for transformation. A planned approach to transformation based on a theory of change, devoting time and attention to skill and relationship building and centering equity, ensuring the active engagement and leadership of community members and creating space for dialog and conversation were common ingredients across all communities. Trust within and across SCALE communities, and the ability to interact and learn were vital. Fostering collaboration and trust needed sustained coaching and technical assistance resources, both to build leadership and improvement skills and to recruit and engage community champions. The reliance on grant funding to support these activities was a limitation.

### 4.1 Contribution to the literature

What does it mean to be in a community system that is truly engaged in learning to advance health, well‐being, and equity in sustainable ways? Results from our evaluation align with the existing literature related to multisector coalitions. Well‐known frameworks such as Collective Impact^21^ emphasize the need for a common agenda, mutual reinforcement, communication and a support system, all of which were also key drivers of success in SCALE. Our results also align with the principles of Collaborating for Equity and Justice^22^ reflecting themes of an explicit focus on equity and racism, sharing power with communities, focusing on systems change and acknowledging complexity. A recent paper by Lardier^23^ echoes our finding that community residents need a psychological sense of community before they choose to engage with coalitions. Wallerstein et al^24^ highlight the need for long term partnerships in participatory research, reflecting our findings of the importance of relationships and trust in facilitating change.

Our evaluation results also advance the knowledge about how community coalitions can build capacity to bring about change. The CLD and the transformation pathways extend the literature beyond just the factors that influence community capacity for change to hypotheses about the *processes* through which these capacities are developed. Even though these pathways were inductively constructed from the unique experiences of individual communities, we discovered enough commonality across communities to have confidence that Figures 2 and 3 are credible processes by which cross‐sectoral community coalitions approach health and well‐being.

### 4.2 Implications for future research

Our findings from SCALE about the dynamics that affect community change also allow us to set a direction for future research. Future efforts must focus on supporting community coalitions to reduce their reliance on external funding and technical assistance for their transformation initiatives. One possibility could be through strengthening regional hubs of excellence with local expertise that enable communities to learn from and support each other. SCALE began to build the infrastructure for regional change by requiring communities to spread the capabilities they had acquired, but in an environment where the communities had competing priorities, limited resources for implementation and staff turnover, there were limitations to what could be achieved. Part of the national and research conversation about shifting power to advance equity and dismantle racism should include ways to shift research and implementation resources to communities.

**FIGURE 4:**
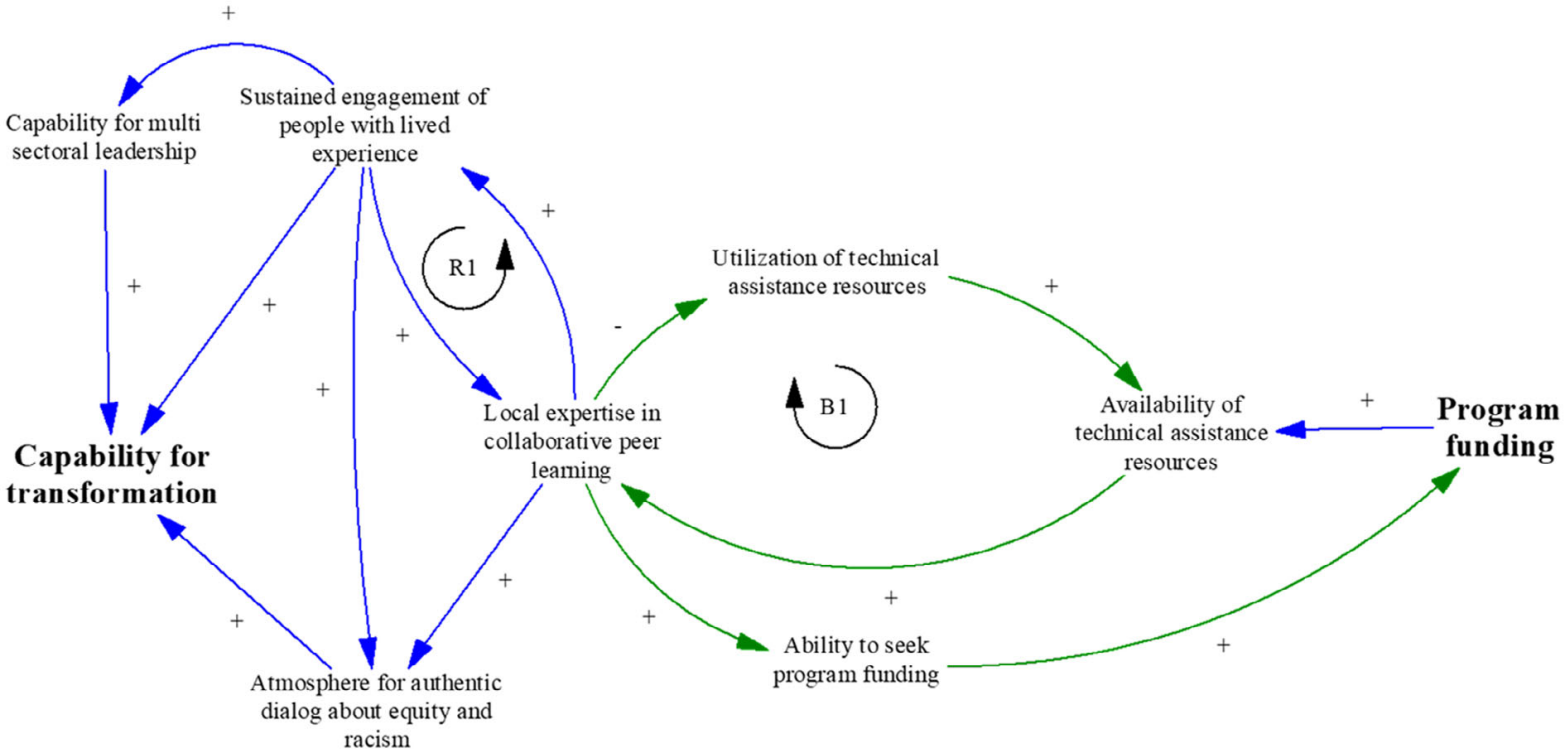
Hypothetical future case scenario

Future research should investigate how to convert the constraints shown in Figure 3 into opportunities. This is illustrated in a hypothetical future system shown in Figure 4. In this figure, the red constraint links in Figure 3 have become green opportunity links. As local expertise increases, the routine need for external technical assistance diminishes, leaving resources available to build specific targeted expertise as needed. This reduces the drain on program funds. At the same time, as communities increase their own ability to acquire funds, the overall availability of funds continues to increase. This enables communities to move from scarcity to abundance, which is a key component of the SCALE theory of change.

## 5 LIMITATIONS

Our evaluation approach was designed to make the best use of existing project data, which was recorded in a variety of systems with varying degrees of completion. Consistent availability of high‐quality data to meaningfully document progress and learning was an ongoing challenge. The SCALE implementation teams introduced multiple measurement plans and mechanisms for data collection over the duration of the initiative, but in the end, there were still gaps in data quality and completeness. The ToAST process used the collective knowledge of the various stakeholder groups and triangulated data from various sources to develop the concepts and transformation pathways, but there were gaps in data that might affect the final results and conclusions. Also, our results have demonstrated progress in skills and behaviors, which are the first two components of theory of change. Given the range and complexity of the projects undertaken by the SCALE communities, there was not enough time for communities to demonstrate how this progress resulted in the achievement of the COH outcomes related to health, well‐being and equity before the funding ran out. This is not unusual, and our inability to evaluate whether the capabilities acquired by the communities resulted in improved health and well‐being is a serious limitation. The second CLD we have presented describes the scenario where communities have sustained financing to continue their transformation efforts. If this scenario were realized, it would be possible to follow communities over time to evaluate the impact of their work. Financial sustainability should be emphasized as a required component for future grants to facilitate this scenario.

## 6 CONCLUSION

The United States has invested trillions of dollars in health, well‐being and equity with little to show for these efforts. Simply investing more money and resources will not likely result in measurable improvement. Complexity‐informed approaches that approach well‐being through the lens of learning rather than through the lens of technical program implementation are needed. SCALE is an example of such an approach. Communities learned how answers to complex questions about change are not apparent at the start of the work, and that practices of learning and testing with meaningful measures to guide progress will help uncover solutions. Learning in a context of complexity requires a willingness to “fail forward,” in an environment where failures are opportunities for learning and growth rather than for judgment. Our results validate the relevance of the elements of the SCALE COS Theory of Change as the key components of skills and behaviors needed to bring about change. The extent to which the acquisition of these skills and behaviors drive sustainable health, well‐being and equity outcomes they aim to achieve should be a priority for future research.

## CONFLICT OF INTEREST

The authors have no conflicts of interest to declare.
